# Psychometric Validation of the Spanish Gaming Disorder Test (GDT): Item Response Theory and Measurement Invariance Analysis

**DOI:** 10.1007/s11469-021-00704-x

**Published:** 2021-12-06

**Authors:** Laura Maldonado-Murciano, Halley M. Pontes, Maite Barrios, Juana Gómez-Benito, Georgina Guilera

**Affiliations:** 1 Faculty of Psychology, University of Barcelona, Passeig de la Vall d’Hebron, 171, 08035 Barcelona, Spain; 2 Institute of Neurosciences, University of Barcelona, Barcelona, Spain; 3 Department of Organizational Psychology, Birkbeck, University of London, London, UK

**Keywords:** Gaming Disorder, Internet Gaming Disorder, Gaming Disorder Test, Item Response Theory, Measurement Invariance

## Abstract

Gaming Disorder (GD) has been recently added to the eleventh revision of the *International Classification of Diseases* (ICD-11) by the World Health Organization (WHO), as such, psychometrically sound psychological measures are required to assess this disorder. The objective of this study was to investigate the psychometric properties of the Spanish Gaming Disorder Test (GDT) by assessing its dimensionality, reliability, convergent validity, and associations with other variables of importance through polytomous Item Response Theory (IRT) and Measurement Invariance (MI) analysis across genders. To achieve this, a sample of 538 gamers (42.94% female, mean_age_ = 23.29 years, SD = 7.24) was recruited. The results obtained supported a one-factor structure for the Spanish GDT with adequate reliability and convergent validity. Furthermore, satisfactory goodness of fit in the partial credit model (PCM) with more precise scores at high trait levels to assess GD was found, and strict invariance across genders was supported. These findings attest to the suitability of the Spanish GDT for clinical assessment and research on disordered gaming beyond community samples.

**Supplementary Information:**

The online version contains supplementary material available at 10.1007/s11469-021-00704-x.

Electronic gaming has become widespread and popular worldwide, playing a major role in the leisure and social pursuits of children, adolescents, and adults (Paulus et al., 2018; Pontes, 2018). The recent figures suggest that there are more than 214 million gamers across the United States (US; Entertainment Software Association, 2020), and about 51% of the European population plays video games (Europe’s Video Game Industry, 2019). In Spain for example, about 15 million people actively played video games in 2019 (Spanish Video Game Association, 2020), further supporting the pervasiveness of gaming in this day and age.

Given the rise in popularity of gaming in today’s society, a key area of research within psychology and psychiatry is related to the potential positive (see Griffiths (2019) and Mandryk et al. (2020)) and negative effects stemming from electronic games, with several studies reporting a wide range of detrimental effects elicited by video game play behaviors, such as aggression (Lemmens et al., 2011; López-Fernández et al., 2020), addiction (Pontes, 2018; Pontes & Griffiths, 2014), and other resulting psychiatric comorbidities (Pontes, 2018; Sherry, 2001), including but not limited to attention-deficit/hyperactivity disorder (ADHD) (Stavropoulos et al., 2019), autism spectrum disorders (ASD) (Craig et al., 2021), and depression (Ostinelli et al., 2021), among other behavioral addictions (Rozgonjuk et al., 2021), with recent evidence linking disordered gaming with poor physical health through impaired psychological well-being (Moore et al., 2021).

Due to its well-documented addictive and detrimental effects (see Männikkö et al. (2020) and Pontes (2017)), the American Psychiatric Association (APA) identified “Internet Gaming Disorder” (IGD) as a tentative disorder in the 5^th^ revision of the *Diagnostic and Statistical Manual of Mental Disorders* (DSM-5; APA, 2013) in 2013. Following this, in 2019 the World Health Organization (WHO) included “Gaming Disorder” (GD) in the 11^th^ revision of the *International Classification of Diseases* (ICD-11; WHO, 2019), further recognizing disordered gaming as an official mental health disorder. Arguably, the inclusion of IGD in the DSM-5 was the first key milestone for research examining the addictive effects of video games, but the culmination of scientific effort regarding its conceptualization and legitimacy was formalized by the inclusion of GD in the ICD-11 and its official recognition as an addictive disorder by the WHO (Pontes & Griffiths, 2020).

Taking this into account, the APA has specified that IGD may be present upon the endorsement of at least five of the nine following diagnostic criteria within a 12-month timeframe: (1) preoccupation with gaming; (2) withdrawal symptoms when gaming is taken away; (3) tolerance, leading to greater amounts of time gaming; (4) unsuccessful attempts to control gaming involvement; (5) loss of interest in previously enjoyed activities as a result of, and with the exception of games; (6) continued excessive gaming behavior despite awareness of problems; (7) deception of family members, therapists, or significant others regarding the amount of gaming; (8) gaming in order to escape or relieve negative moods; and (9) jeopardizing or losing a significant relationship, job or education, or career opportunity because of participation in games (APA, 2013).

Moreover, the WHO further conceptualized GD by suggesting that it can occur both online and/or offline and that GD requires the experience of (1) diminished control in relation to gaming (e.g., onset, frequency, intensity, duration, termination, and context), (2) an increase in the priority given to gaming to a point that it takes precedence over other important life interests and activities, and (3) continuation or escalation of gaming regardless of the experience of detrimental consequences. Furthermore, the gaming behavior pattern should be of sufficient severity to result in significant impairments in personal, family, social, educational, occupational, or other important areas of life (WHO, 2019).

## The Current Study

Since the earlier inclusion of IGD by the APA in the DSM-5 as a tentative mental health disorder, several psychometric tests have been developed to assess disordered gaming based on the APA framework (Pontes et al., 2021), among which a few have been translated and psychometrically validated to Spanish-speaking samples. Specifically, the Internet Gaming Disorder Test (IGD-20 Test; Pontes & Griffiths, 2014), the Internet Gaming Disorder Scale-Short Form (IGDS9-SF; Pontes & Griffiths, 2015), and the Ten-Item Internet Gaming Disorder Test (IGDT-10; Király et al., 2017) have all been tested in Spanish-speaking populations, with studies generally reporting promising psychometric properties (Beranuy et al., 2020; Fuster et al., 2016; Király et al., 2019; Maldonado-Murciano et al., 2020; Sánchez-Iglesias et al., 2020). Although these standardized psychological measures are useful and psychometrically sound for assessing disordered gaming (Poon et al., 2021), they have been developed to assess IGD under the APA framework. Therefore, they do not fully take into account the latest conceptualization of GD proposed by the WHO in the ICD-11, making it necessary to further investigate and refine the psychometric assessment of GD under the WHO framework (Montag et al., 2019).

To bridge this gap in the assessment of disordered gaming, a brief standardized psychological measure including four items reflecting the key defining diagnostic features of GD according to the WHO framework has been recently developed (Pontes et al., 2021). The Gaming Disorder Test (GDT) was originally developed in English and Chinese-speaking samples (Pontes et al., 2021) due to GD being an emerging public health concern in Asia and developed Western countries (Evren et al., 2020). The GDT has been subsequently psychometrically validated and adapted in German (Montag et al., 2019) and Turkish samples (Evren et al., 2020), with the findings of these recent studies suggesting that the GDT presents with robust psychometric properties and that it is a suitable psychological measure to assess GD across several populations according to the WHO framework.

Based on the aforementioned rationale, the goal of the present study was to develop the first Spanish version of the GDT through Classical Test Theory (CTT) and Item Response Theory (IRT) in order to report its psychometric suitability to assess GD under the WHO framework within the Spanish population. By achieving this goal, this study will be contributing to the field by providing additional information on the suitability of the WHO framework to assess disordered gaming within an international context (i.e., non-English context), adding to the knowledge base about the assessment of GD while further providing a practical resource to health professionals in Spain to assess the severity of GD within the Spanish cultural context.

## Methods

### Participants and Procedures

A sample of Spanish gamers was recruited using two inclusion criteria (i.e., being at least 16 years and having played a video game once in their lifetime). The study was conducted in accordance with the Declaration of Helsinki and approved by the Committee on Bioethics of the University of Barcelona.

Participation was voluntary, and no financial compensation was offered to eligible participants. An online informed consent was obtained from all participants after they had been informed about the anonymous and confidential nature of the study. Data collection was conducted using an online survey hosted on Qualtrics, which included questions assessing participants’ sociodemographic status, gaming behaviors, personality traits, and psychiatric symptoms. Data collection spanned from April 15 to November 6, 2020, and the survey was advertised online via multiple social media platforms (i.e., Facebook, Instagram, Reddit, and Twitter) and on the online course management system of a second-year course of the degree of Psychology of the University of Barcelona.

A sample of 569 participants was initially recruited. However, a total of 31 participants were excluded from the study for presenting with missing data (*n* = 21, 3.69%) or for declaring being a professional video game player (*n* = 10, 1.76%). Since no additional participants were removed due to missing data, a final sample of 538 participants was achieved and their data were subsequently included in the statistical analyses conducted.

Within the final sample, a 42.94% (*n* = 231) of participants were female (age range: 18–57 years) and 57.09% (*n* = 307) were males (age range: 18–56 years). The overall mean age was 23.29 years (*SD* = 7.24; range: 16–57 years). Moreover, most participants completed a secondary educational level (55.76%, *n* = 300) or a higher educational level (30.85%, *n* = 166). In relation to gaming behaviors, participants reported having played an average of 1.98 hour a day on working days (*SD* = 2.07; range: 0–16 hours) and 3.48 hours during non-working days (*SD* = 2.86; range: 0–16 hours).

### Measures

#### Sociodemographic Data

The survey collected sociodemographic data aligned with previous similar psychometric studies (e.g., Maldonado-Murciano et al., 2020). More specifically, data were collected about participants’ gender, age, educational level achieved, and gaming-related behaviors (e.g., time spent gaming during the working days and non-working days such as weekends and holidays).

#### Gaming Disorder Test (GDT) (Pontes et al., 2021)

The GDT is a brief 4-item standardized psychological test assessing disordered gaming according to the WHO framework, adopting the proposed conceptualization for GD by the WHO in the ICD-11. The first three items of the GDT reflect (1) *impaired control over gaming*, (2) *increased priority given to gaming*, and (3) *continuation despite negative consequences*, while the last and fourth item assesses potential functional impairments by evaluating gamers’ (4) *experience of significant problems in life* due to GD.

Responses to all four GDT items can be given on a 5-point scale ranging from 1 (*never*) to 5 (*very often*). Total scores can range from 4 to 20 points, with higher scores indicating greater degrees of disordered gaming. For non-clinical purposes, answers given to all four GD items as 4 (*often*) or 5 (*very often*) can be coded to reflect endorsement of a specific GD criterion.

The Spanish version of the GDT was devised by adopting a double-translation and reconciliation procedure for the translation of the original English items of the GDT. This procedure involved two psychologists who independently translated the original GDT items from English into Spanish. Following this, a third independent translator identified and resolved any discrepancies between the alternative Spanish forward translations generated (International Test Commission, 2018). Interested readers can access the final Spanish version of the GDT in Appendix, with further information about the GDT being presented in the author's website (www.halleypontes.com/gdt).

#### Internet Gaming Disorder Scale-Short Form (IGDS9-SF) (Pontes & Griffiths, 2015)

The IGDS9-SF is a 9-item standardized test used to assess disordered gaming as per the APA framework for IGD defined in the DSM-5. All nine items can be responded to using a 5-point response scale ranging from 1 (*never*) to 5 (*very often*), and greater overall scores indicate higher levels of disordered gaming symptoms. The IGDS9-SF has been shown to present with robust psychometric properties according to a recent review study of 21 studies across 15 languages that employed the IGSD9-SF (Poon et al., 2021). Spanish IGDS9-SF has been found to present with excellent psychometric properties under the CTT (Beranuy et al., 2020; Sánchez-Iglesias et al., 2020) and IRT frameworks (Maldonado-Murciano et al., 2020). In the present study, the IGDS9-SF exhibited high levels of internal consistency (*α* = 0.90 and *ω* = 0.85).

#### Mini International Personality Item Pool-Five-Factor Model-Positively Worded (Mini-IPIP-PW) (Donnellan et al., 2006)

The Mini-IPIP-PW was used to evaluate personality traits under the five-factor model of personality (Goldberg, 1992). These include the traits extraversion, agreeableness, conscientiousness, neuroticism, and openness to experience. The test comprises 20 items answered using a 5-point response scale response ranging from 1 (*totally disagree*) to 5 (*totally agree*). In the present study the Spanish positively worded version was utilized (Bados López et al., 2005) as it has been shown to have high levels of reliability, convergent, and predictive validity (Martínez-Molina & Arias, 2018). The Spanish Mini-IPIP-PW has exhibited high levels of internal consistency across its different domains in the present sample (see Table 1).

**Table 1:** Descriptive statistics of the IGDS9-SF, the MINI-IPIP-PW, and the DASS-21 and their correlations with the Gaming Disorder Test (GDT)

Measure	Mean	*SD*	*α*	*ω*	*r*
IGDS9-SF (*n* = 538)	14.44	5.39	.90	.85	.76**
Mini-IPIP-PW (*n* = 538)					
Neuroticism	10.77	3.61	.79	.77	− .01
Extraversion	10.81	3.83	.82	.80	− .04
Agreeableness	15.64	3.13	.90	.86	− .16**
Openness	13.89	3.57	.82	.88	.10*
Consciousness	12.59	3.35	.80	.81	− .24**
DASS-21 (*n* = 276)					
Anxiety	8.80	9.38	.90	.85	.08
Depression	14.88	11.27	.93	.91	.23**
Stress	14.52	9.62	.88	.86	.14*

*SD* standard deviation *α* Cronbach’s alpha coefficient, *ω* omega coefficient, *p < .05 **p < .01, *IGDS9-SF* Internet Gaming Disorder Scale-Short Form, *MINI-IPIP-PW* Mini-International Personality Item-Pool-Five-Factor Model-Positively Worded, *DASS-21* Depression, Anxiety, and Stress Scales

#### Depression, Anxiety, and Stress Scales (DASS-21) (Lovibond & Lovibond, 1995)

The DASS-21 includes 21 items that can be responded to on a 4-point Likert scale ranging from 0 (*did not apply to me at all*) to 3 (*applied to me very much, or most of the time*). The DASS-21 is used to assess psychiatric symptoms of depression, anxiety, and stress. The Spanish DASS-21 has been shown to exhibit adequate internal consistency, satisfactory convergent validity, and acceptable discriminant validity (Bados López et al., 2005). In the present study, the Spanish DASS-21 has exhibited high levels of internal consistency across its different domains (see Table 1).

### Data Analysis

For the purpose of describing the distribution of the GDT items’ scores, the frequency of endorsement of each item response category and item skewness and kurtosis were obtained. Similarly, for the GDT total score, general descriptives and the Shapiro–Wilk test (*W*) of univariate normality were computed for overall sample. The Mardia test was also utilized to assess multivariate normality across the GDT items.

In order to assess the one-factor structure of the Spanish GDT, a Confirmatory Factor Analysis (CFA) was estimated using the Weighted Least Square Mean and Variance Adjusted (WLSMV), which has been found to provide accurate parameter estimates with ordinal items, whereby items are rated with few response categories, in relatively small sample sizes, and when departures from multivariate normality are observed (Li, 2016). The model fit was assessed with the Comparative Fit Index (CFI), the Tucker-Lewis Index (TLI), the Root Mean Square Error Approximation (RMSEA), and the Standardized Root Mean Square Residual (SRMR). Goodness of fit was interpreted using the recommended guidelines proposed by Hu and Bentler (1999) where an adequate fit was observed when CFI ≥ 0.95, TLI ≥ 0.95, RMSEA ≤ 0.06, and SRMR ≤ 0.08.

The Average Variance Extracted (AVE) coefficient for the GD factor was also estimated. Reliability was additionally assessed using different indicators (i.e., Cronbach’s alpha (*α*), McDonald’s omega (*ω*), and Composite Reliability (CR)). Moreover, validity based on relationships with other relevant variables was assessed by computing Pearson correlation coefficients between the GDT and the other relevant psychometric tests used in the study for measuring GD, personality traits, and psychiatric symptomatology (i.e., IGDS9-SF, MINI-IPIP-PW, and DASS-21, respectively).

In order to complement the CTT analyses performed, a follow-up IRT analysis was conducted on the Spanish GDT as IRT models produce useful information about the quality of items and provide measures of precision at different levels of the trait ($\theta$) (Embretson & Reise, 2000). Previously to the IRT analysis, local independence and unidimensionality assumptions were respectively inspected, computing Yen’s *Q*_3_ statistic, with critical values of item residual correlations > 0.20 indicating local dependence (Chen & Thissen, 1997; Christensen et al., 2017) and fitting a one-factor model by the CFA formerly described. Due to the ordinal nature of the four items, we compared the model fit of three competing models: the Partial Credit Model (PCM) (Masters, 1982), the Generalized Partial Credit Model (GPCM) (Muraki, 1992), and the Graded Response Model (Samejima, 1999). The fit of the models was compared using the Bayesian Information Criteria (BIC) and Akaike Information Criteria (AIC), selecting the model with lower values, which indicates closer fit to the true model (Burnham & Anderson, 2004).

For the best fitting model, we calculated the item fit parameters using S-*χ*^2^ statistic and the INFIT and OUTFIT (Wright & Panchapakesan, 1969), the Z_h_ person fit statistic (Drasgow et al., 1985), the items’ Operating Characteristic Curves (OCC), and the information function of both the items and the test. Potential item misfit is indicated by statistically significant values of the S-*χ*^2^ statistic (Kang & Chen, 2008; Orlando & Thissen, 2000) and by INFIT and OUTFIT values less than 0.5 or greater than 1.5 (de Ayala, 2009), while values of the Z_h_ statistic ≤  − 2 suggest person misfit (Desjardins & Bulut, 2018).

Additionally, Measurement Invariance (MI) of the Spanish GDT across gender was investigated. MI analysis is grounded on the notion that a psychometric test measuring a given trait should reveal differences among individuals if those individuals actually differ on the trait (Millsap, 2011). Thus, if we intend to use GDT scores to make comparisons among male and female gamers, we must then ensure that the latent variable (i.e., GD) is functioning similarly across the two groups of participants. The evaluation of gender invariance was carried out sequentially assessing (1) configural invariance, to investigate the equivalence of the factor structure; (2) weak or metric invariance, to test equivalence of the item loadings on the latent factor; (3) strong or scalar invariance, to test the equivalence of intercepts; and (4) strict or residual invariance, to assess the equivalence of residual variances (Desjardins & Bulut, 2018).

According to Sass et al. (2014), since interpreting changes in approximate fit indices may be controversial with diagonal weighted least squares-based estimators, the comparison between MI models was based on the examination of chi-square difference (Δ*χ*^2^) and its corresponding statistical significance. Finally, since a non-normal distribution of the GDT total scores was observed, the non-parametric test Mann–Whitney *U* test was adopted in order to estimate gender differences on GDT total scores.

All statistical analyses were conducted with R (version 1.0.136) (R Core Team, 2021), using the packages *lavaan* (Rosseel, 2012) for the CFA and MI analysis, *semTools* (Jorgensen et al., 2020), and *mirt* (Chalmers, 2012) for the IRT analysis.

## Results

### Distribution of GDT Scores

As shown in Table 2, most of the participants’ responses for all items are ubicated in the first or second response category (*never* or *rarely*, respectively), with very few participants endorsing the fifth category (*very often*). Skewness and kurtosis coefficients suggested that item 4 was right-skewed, with almost 80% of participants endorsing the lowest item response category (*never*). In terms of univariate normality testing, results showed that the GDT total score was positively skewed (*W* = 0.879, *p* < 0.001), indicating a tendency to lower levels of GD (*M* = 7.04, *SD* = 2.98, range: 4–17, *Md* = 2.97). As for the multivariate normality assessment, the results of the Mardia test found that the data was not normally distributed (skewness = 879.901, *p* < 0.001; kurtosis = 26.018, *p* < 0.001) Table3 and 4.

**Table 2:** Endorsement, kurtosis, and skewness of GDT items

Item	Frequency of item endorsement (%)					Skewness	Kurtosis
	1	2	3	4	5		
1	240 (44.61)	192 (35.69)	77 (14.31)	24 (4.46)	5 (0.91)	1.04	.67
2	220 (40.89)	141 (26.21)	126 (23.42)	44 (8.17)	7 (1.30)	.65	− .54
3	255 (47.40)	132 (24.54)	100 (18.59)	44 (8.18)	7 (1.30)	.87	− .25
4	429 (79.74)	76 (14.13)	24 (4.46)	7 (1.30)	2 (0.37)	2.64	7.65

**Table 3:** Item statistics for the Partial Credit Model (PCM) across the four items of the Spanish Gaming Disorder Scale (GDT)

Item	*β*_1_	*β*_2_	*β*_3_	*β*_4_	S-*χ*^2^	*df*	*p*	*Infit*	*Outfit*
1	− 0.16	2.03	3.53	4.95	7.21	10	.705	0.803	0.686
2	− 0.18	0.83	2.97	4.91	11.41	11	.410	0.742	0.630
3	0.22	1.16	2.84	4.94	37.6	13	< .001	0.735	0.597
4	2.32	3.34	4.45	5.21	17.85	10	.058	0.863	0.606

*β* difficulty parameter, *S-χ*^*2*^ generalized chi-square statistic (Kang & Chen, 2008; Orlando & Thissen, 2000), *df* degrees of freedom, *p* chi-square significance value

**Table 4:** Gender measurement invariance indices of the Spanish GDT

Model	*χ*^2^(df)	CFI	RMSEA	Δ*χ*^2^(*df*)	*p*(Δ*χ*^2^)
Configural	2.210 (4)	0.982	0.077	-	-
Metric (weak)	5.412 (7)	0.984	0.054	4.186 (3)	.242
Scalar (strong)	7.873 (10)	0.978	0.053	5.029 (3)	.170
Strict	9.916 (14)	0.983	0.039	3.735 (4)	.443

*df* degrees of freedom, *CFI* comparative fit index, *RMSEA* root mean square error of approximation

### Dimensionality

**Fig. 1:**
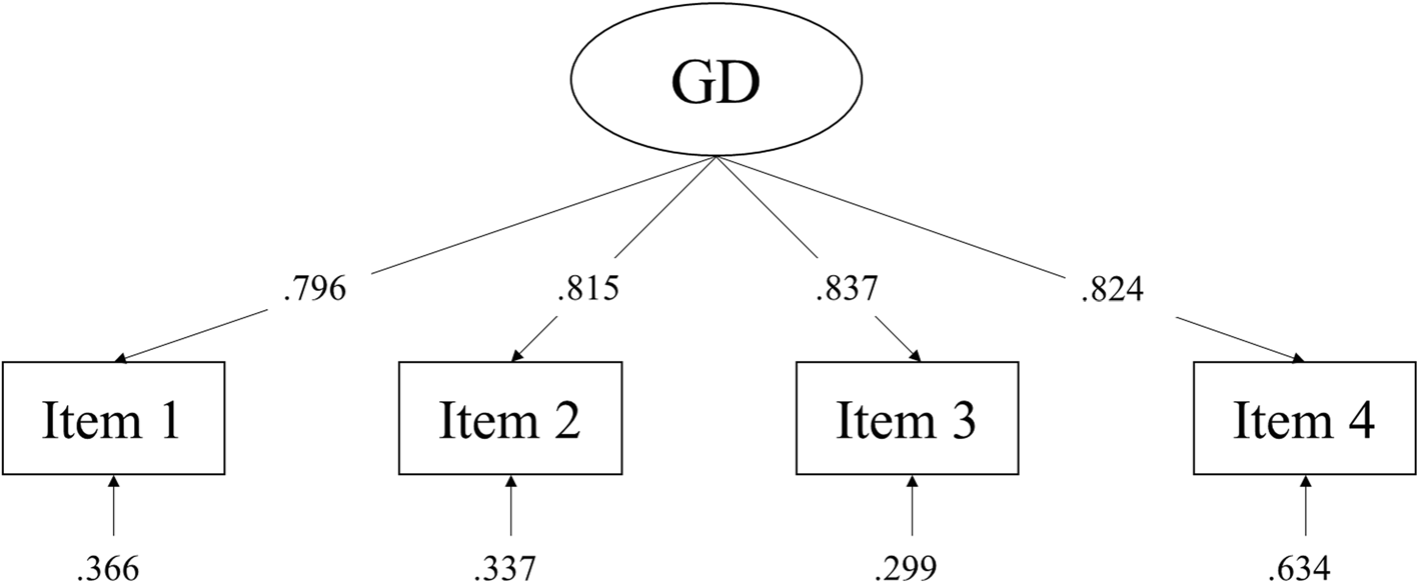
Path diagram with summary of the confirmatory factor analysis (CFA) obtained from the four items of the Gaming Disorder Test (GDT). Notes: GD, gaming disorder

A CFA was carried out on the four items of the Spanish GDT in order to test the unidimensionality of the scale. The results obtained supported a one-factor solution (*χ*^2^(2) = 3.847, *p* = 0.146; CFI = 0.999; TLI = 0.997; RMSEA = 0.041 [90% CI: 0.000–0.104], *p* = 0.489; SRMR = 0.016). The path diagram (see Fig. 1) shows that all standardized factor loadings were high and statistically significant (*λ* > 0.750, *p* < 0.001).

### Reliability

The AVE is used as evidence of adequate convergence between items of a psychometric test when its value is ≥ 0.50 and CR is ≥ 0.70 (Hair et al., 2010). The GDT obtained an AVE of 0.669 and CR of 0.890. Moreover, further evidence supporting the reliability of the Spanish GDT was obtained with a Cronbach’s alpha of *α* = 0.889 and a McDonald’s omega of *ω* = 0.839. Based on these results, it can be concluded that the Spanish GDT presents with adequate levels of convergence between items and reliability.

### Concurrent, Convergent, and Discriminant Validity

As can be seen in Table 1, the GDT was strongly associated with the IGDS9-SF (*r* = 0.76). In relation to the personality and psychiatric symptoms scores, the GDT moderately correlated with the depression subscale (*r* = 0.23) and with consciousness (*r* =  − 0.24), respectively. Furthermore, a weak association was observed between GDT and agreeableness (*r* =  − 0.16), openness (*r* = 0.10), and stress (*r* = 0.14). Taken together, these findings provide adequate evidence of concurrent, convergent, and discriminant validity for the Spanish GDT.

### IRT Analysis of the Spanish GDT

Unidimensionality and local independence assumptions were met as shown in the CFA and the results of the Yen’s *Q*_3_ statistic, which indicated that residual correlations ranged between − 0.383 and − 0.162. The AIC (AIC_PCM_ = 4137.917; AIC_GPCM_ = 4143.280; AIC_GRM_ = 4139.131) and the BIC (BIC_PCM_ = 4210.810; BIC_GPCM_ = 4229.037, BIC_GRM_ = 4325.016) values obtained suggest that the IRT model with best fit to the data was the PCM. Table 3 shows the item difficulty (*β*) parameters and item fit statistics.

**Table 5:** Descriptive statistics and reliability indices of the Gaming Disorder Test (GDT) in male and female participants

	Female (*n* = 231)				Male (*n* = 296)			
Measure	*M*	*SD*	*α*	*ω*	*M*	*SD*	*α*	*ω*
GDT	6.78	2.71	.776	.796	7.19	3.12	.835	.848

*M* mean, *SD* standard deviation, *α* Cronbach’s alpha coefficient, *ω* omega coefficient

Altogether, the item threshold parameters covered a wide range of the latent trait, especially the middle-upper band (i.e., ranging from − 0.16 to 5.21), suggesting that a high latent trait level is needed to endorse high item response categories. The higher values observed in item 4 (i.e., *I have experienced significant problems in life (e.g., personal, family, social, education, occupational) due to the severity of my gaming behavior*) are indicating that this is the most difficult item to endorse (i.e., high level of GD is needed to endorse the high item response categories). Conversely, item 1 (i.e., *I have had difficulties controlling my gaming activity*) was the easiest item to endorse (i.e., low level of GD is needed to endorse high item response categories). Regarding item fit based on S-*χ*^2^ statistic, results indicated that GDT items 1, 2, and 4 presented with adequate fit to the PCM model, while item 3 exhibited poor fit.

**Fig. 2:**
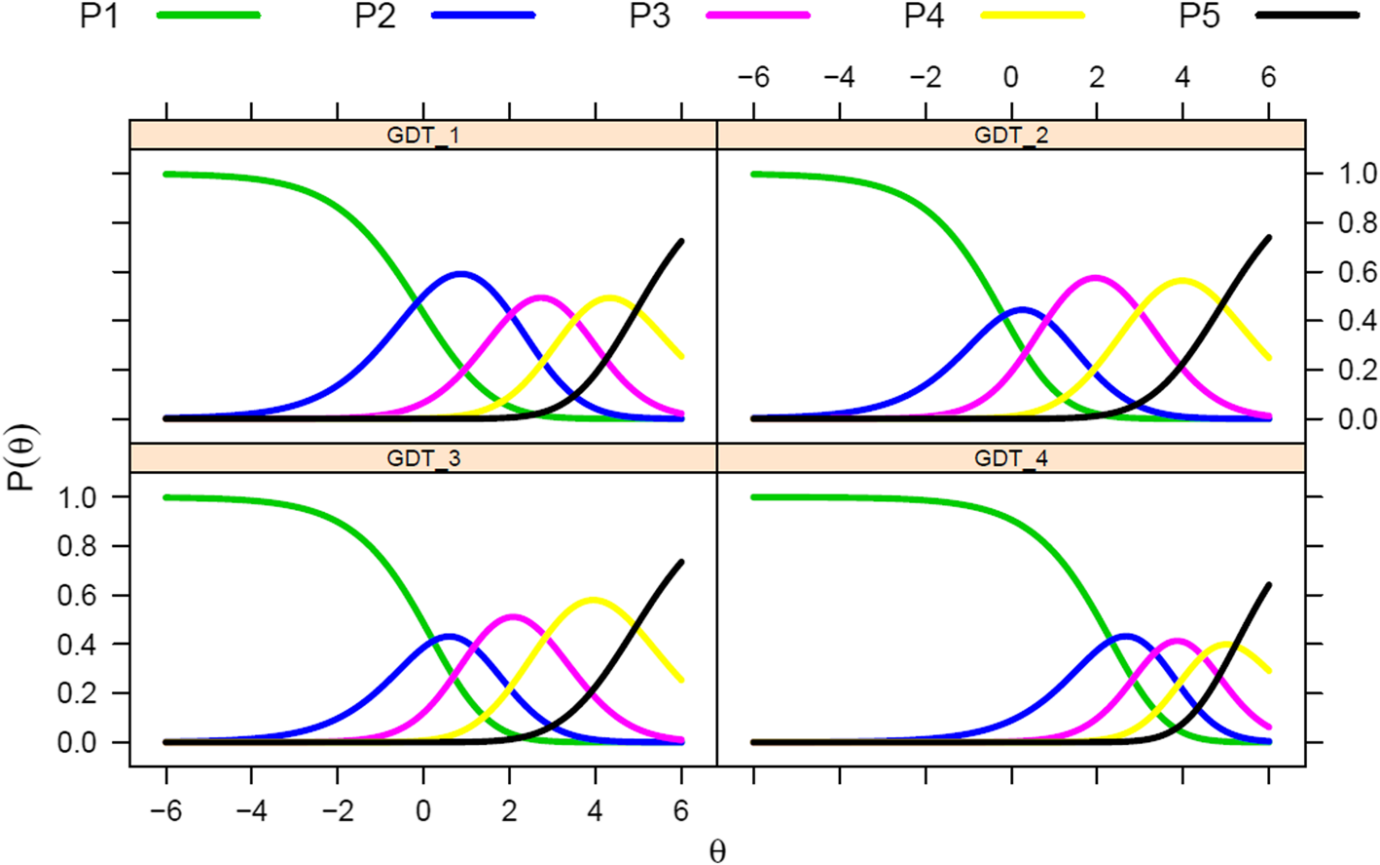
Operating Characteristic Curves (OCC) of the four items of the Gaming Disorder Test (GDT)

A visual inspection of the empirical plot for item 3 (see Fig. S1) suggested that the misfit was due to discrepancies between the theoretical model and the empirical data on response categories 2 (*rarely*) and 3 (*sometimes*). However, since the S-*χ*^2^ statistic is very sensitive to sample size (Jöreskog, 1993), items’ INFIT and OUTFIT values were inspected showing that all values were within the recommended range (0.5 ≤ INFIT/OUTFIT ≤ 1.5). To further assess the model fit, person fit indices and items’ OCCs were calculated (Embretson & Reise, 2000). Person fit Z_h_ statistic showed that 98.33% of participants’ response patterns were aligned with the PCM (see Fig. S2). In addition, the items’ OCCs (Fig. 2) indicated that all four items’ response categories of the Spanish GDT were ordered according to increasing levels of the latent variable and did not overlap between them, demonstrating the suitability of the 5-point response scale of the Spanish GDT items.

**Fig. 3:**
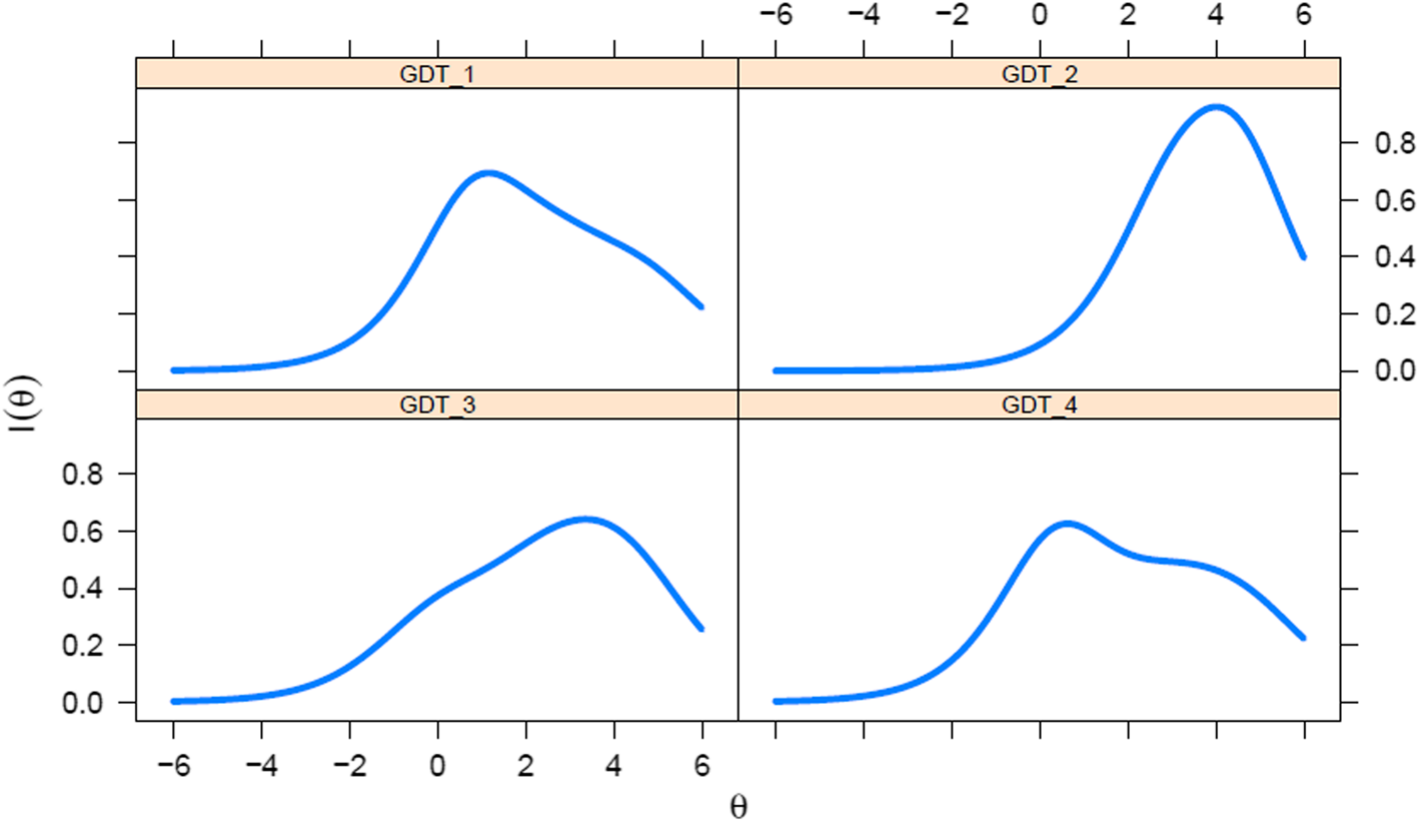
Item information curves of the four items of the Gaming Disorder Test (GDT)

The item information function shows the amount of information that each item explains as a function of the latent trait level. As shown in Fig. 3, item 1 (i.e., *impaired control over gaming*) and item 4 (i.e., *experience of significant problems in life*) were more informative at the medium levels of the latent trait (i.e., *θ* close to 0). In contrast, item 2 (i.e., *increased priority giving to gaming*) was more informative at higher levels of the latent trait (i.e., peak of precision around *θ* = 3.5) and to a greater extent item 3 (i.e., *continuation despite negative consequences*) was more informative even at higher levels of the latent trait (i.e., peak of precision around *θ* = 4).

**Fig. 4:**
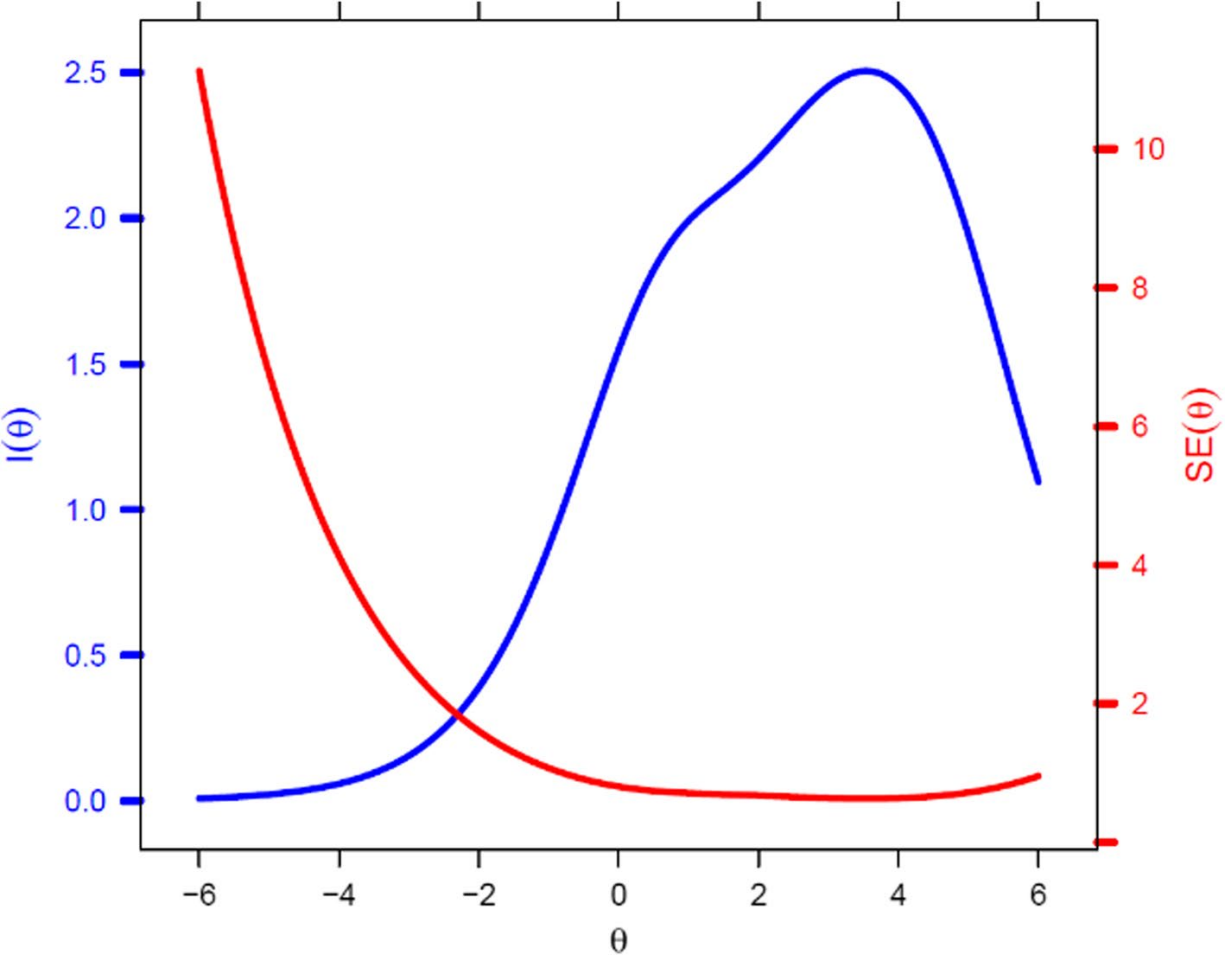
Test information curve and standard error of the Gaming Disorder Test (GDT)

The test information function and standard error (Fig. 4) revealed that the test as a whole was more informative at the highest levels of the trait, more specifically when the trait remains between 1 and 5, and is less precise at lower levels of the latent trait (i.e., θ < 0).

### Measurement Invariance Between Genders

The GDT MI between genders was limited to male and female participants, leaving aside those who identified themselves with another gender due to the low sample size in this particular group (*n* = 11, 2.04%). As shown in Table 4, the chi-square difference statistic was not statistically significant across the configural, metric, scalar, and strict invariance models. This suggests that the Spanish GDT presents strict invariance, where loadings, intercepts, and residual variances are invariant between males and females.

The GDT total score was compared between males and females with the Mann–Whitney *U* test, suggesting that there were no differences between genders (*U* = 32,364, *p* = 0.287). Descriptive statistics and reliability indices of the GDT total score are shown in Table 5 for both gender groups.

## Discussion

The present study sought to adapt the GDT into Spanish and to conduct a psychometric validation using both CTT and IRT analytical frameworks, with the goal of testing the suitability of the GDT for assessing GD within the Spanish context. For this purpose, the English version of the GDT was translated into Spanish following conventional international standards (International Test Commission, 2018), administered to a large sample of Spanish video gamers, and data on psychometric indicators was gathered in a cross-sectional online survey study.

Based on the findings reported, it can be concluded that the GDT is a valid and reliable psychometric test for assessing GD as suggested by the ICD-11 (World Health Organization, 2019). More specifically, the results of the CFA supported the unidimensionality of the Spanish GDT, with high and statistically significant standardized factor loadings. This unifactorial model was found to be gender invariant at the highest level (i.e., strict invariance). This implies that the GD construct as assessed by the GDT is equivalent across males and females and, consequently, GDT scores present the same meaning for both genders, enabling adequate comparisons of the mean GDT total scores between male and female gamers.

This result is encouraging as it is the first gender MI analysis of the GDT in Spanish population. However, further invariance testing assessing other relevant groups (e.g., GD diagnostic invariance or cross-cultural invariance) would provide additional information about the capabilities of the GDT in terms of MI across groups. In contrast to other studies (Arıcak et al., 2018), male and female participants in our study showed similar levels of GD.

The results pertaining to the reliability analysis of the Spanish GDT demonstrated high levels of internal consistency for the overall sample and across genders in terms of the Cronbach’s alpha and omega reliability coefficients. A detailed analysis at the item level by means of IRT indicated that the Spanish GDT items performed adequately and were more informative at medium–high levels of the GD trait. Despite the statistical misfit of item 3 to the PCM, this item was retained given that other indicators analyzed in the present study supported its adequate performance, and its retention balances the content coverage of the GDT as based on the ICD-11 criteria of GD, and other studies with empirical data have shown that the misfit generally implies a negligible practical impact on score estimates (Sinharay & Haberman, 2014; Zhao, 2017). Furthermore, the test information functions revealed that GDT scores were more accurate at middle-upper levels of the latent trait, suggesting that beyond its application in community samples, the GDT may be especially useful in clinical settings where GD symptoms are prominent and the brevity of administration is a priority.

Moreover, the results obtained provided further support for the concurrent, convergent, and discriminant validity of the Spanish GDT. Concurrent validity was supported by the strong correlation between GDT and IGDS9-SF scores, as they reflect the intensity of disordered symptoms. The strength of this association was similar to that obtained in previous studies (Pontes et al., 2021). Furthermore, GDT scores were also moderately associated with depression and weakly associated with stress (convergent validity). Previous research has shown that depression symptoms have been consistently associated with severity of GD (Ostinelli et al., 2021), showing that adults with depression may resort to gaming to escape from adverse emotions (Kim et al., 2017). In relation to the finding pertaining to stress, the weaker association may be due to the intricate relationship between GD and stress responses as stressful life events are an important predictor of disordered gaming (Li et al., 2016) while at the same time GD has been found to be a stress response (Snodgrass et al., 2014).

The correlation between GD and anxiety was surprisingly low in the present study. Previous studies that have evaluated the relationship between GD, as measured by the IGDS9-SF, and anxiety (i.e., measured with the DASS-21) have found a stronger relationship between the two constructs (Pontes & Griffiths, 2016; Yam et al., 2019), suggesting either that anxiety-related symptoms may be better represented in the DSM-5-based instruments such as the IGDS9-SF or that the coronavirus pandemic (COVID-19) may have influenced the results. In relation to the latter, taking into account that data collection of the present study was undertaken during the COVID-19 pandemic, the low correlation between GD and anxiety may be explained by the current pandemic situation as it is likely that baseline levels of mental health factors (e.g., anxiety) among the participants recruited were modulated by the mental and emotional toll brought about the COVID-19 (Liang et al., 2020) potentially confounding the findings related to anxiety.

In terms of personality traits, GDT scores were inversely associated to conscientiousness with a moderate strength. In line with this result, Müller et al. (2014) found that low consciousness was a predictor for addictive disorders, and Dieris-Hirche et al. (2020) concluded that disordered gamers showed lower levels of conscientiousness. To a lesser extent, GD was inversely related to agreeableness and directly to openness, although both associations were very weak. Other studies have found agreeableness to be negatively correlated with motives to play (de Hesselle et al., 2020) and even defined as a protective factor of behavioral addictions (Kayiş et al., 2016) such as GD (Mihara & Higuchi, 2017). Results on openness are contradictory; some studies have found an inverse association and some others a non-existing association (Şalvarlı & Griffiths, 2019). Although neuroticism has been repeatedly associated with GD (Dieris-Hirche et al., 2020; Mihara & Higuchi, 2017; Wittek et al., 2016), this finding was not observed within the present study. Future studies should be conducted in order to disentangle relationships between GD, as measured by the GDT, and other relevant constructs for gathering additional evidence of nomological validity.

Taking together, the results on the validity and reliability allow us to conclude that the Spanish GDT is an adequate psychometric test to assess GD within the Spanish population, reaching similar promising results than those found with the Spanish version of the Spanish IGDS9-SF (Beranuy et al., 2020; Maldonado-Murciano et al., 2020; Sánchez-Iglesias et al., 2020). Since the Spanish IGDS9-SF assesses disordered gaming under the APA framework, the present Spanish GDT represents a better assessment option for health professionals and clinicians alike when conducting assessment of disordered gaming adopting the WHO framework. Moreover, the Spanish GDT offers additional practical benefits due to its brevity.

Despite the findings reported, the present study is not without limitations. One of the main limitations is the sampling strategy used to recruit participants, as participants were self-selected. Consequently, the results reported cannot be directly generalized to the general Spanish population. Further research utilizing different sampling strategies (e.g., random sampling) should be conducted to help overcome the current sampling limitations and estimate prevalence of GD in more representative samples. Since a clinical sample was not recruited to the present study, we were not able to explore the diagnostic accuracy of the Spanish GDT in terms of its sensitivity and specificity nor test its MI across diagnostic groups. Future studies may help advance the literature on the assessment of GD under the WHO framework by examining the diagnostic accuracy of the GDT in clinical samples using a valid and reliable gold standard (i.e., a formal psychiatric assessment) that also allows to estimation of cut-off points for the GDT. Moreover, in the absence of a clinical gold standard, researchers may develop *ad hoc* cut-off points using mixture modeling techniques such as latent profile analysis or latent class analysis to derive an empirical gold standard, similar to what has been done in past research (Fuster et al., 2016; Király et al., 2017; Pontes et al., 2014; Severo et al., 2020).

Finally, it is plausible that decreased mental health levels due to the COVID-19 pandemic may have influenced the relationship between GD and mental health factors investigated in this study. Notwithstanding these potential limitations, the results obtained indicate that the Spanish GDT is a useful psychometric test to assess GD in community-based samples across a wide age range within the Spanish context, lend empirical support for the concept of GD as suggested by the ICD-11, and pave the way for new psychometric research on GD in Spanish-speaking countries.

## Conclusion

This study has developed the Spanish GDT and investigated its psychometric properties, thus contributing to advancing the current understanding of GD and its assessment under the WHO framework. The results reported suggest that the Spanish GDT presents a unidimensional factor structure, consistent test scores, measurement invariance across genders, and that the items of the GDT are more precise at medium and high levels of the GD trait. Notably, the GDT is a promising assessment tool that can be used in both clinical and epidemiological studies within the Spanish context.

### Electronic supplementary material

Below is the link to the electronic supplementary material.

Supplementary file1 (DOCX 94 KB)

## Appendix. The Spanish Gaming Disorder Test (GDT)

**Instrucciones:**

Las siguientes preguntas se refieren a tu actividad de juego con videojuegos durante el último año (es decir, los últimos 12 meses). Por actividad de juego con videojuegos nos referimos a cualquier actividad de juego llevada a cabo desde un ordenador (de sobremesa o portátil) o desde una consola o cualquier otro dispositivo (por ejemplo, móvil, tableta, etc.), tanto de forma online (con conexión a internet) como offline (sin conexión a internet).

Por favor, indique con qué frecuencia han ocurrido los siguientes problemas en promedio durante los últimos doce meses hasta el día de hoy.

**Table:**

	Nunca	Raramente	A veces	A menudo	Muy a menudo
1. He tenido dificultades para controlar mi actividad de juego con videojuegos					
2. He dado mayor prioridad a jugar a videojuegos que a otros intereses vitales y actividades diarias					
3. He continuado jugando a videojuegos a pesar de las consecuencias negativas					
4. He experimentado problemas significativos en mi vida (p.ej., personales, familiares, sociales, educativos o laborales) debido a la gravedad de mi comportamiento de juego con videojuegos					

## References

[CR1] American Psychiatric Association (2013). Diagnostic and statistical manual of mental disorders.

[CR2] Arıcak OT, Dinç M, Yay M, Griffiths M (2018). Adapting the short form of the Internet Gaming Disorder Scale into Turkish: Validity and reliability. Addicta: The Turkish Journal on Addictions.

[CR3] Asociación Española de Videojuegos (2020). La industria del videojuego en España. Anuario.

[CR4] Bados López A, Solanas A, Andrés R (2005). Psycometric properties of the Spanish version of Depression, Anxiety and Stress Scales (DASS). Psicothema.

[CR5] Beranuy M, Machimbarrena JM, Vega-Osés MA, Carbonell X, Griffiths MD, Pontes HM, González-Cabrera J (2020). Spanish validation of the internet gaming disorder scale–short form (IGDS9-SF): Prevalence and relationship with online gambling and quality of life. International Journal of Environmental Research and Public Health.

[CR6] Burnham KP, Anderson DR (2004). Multimodel inference: Understanding AIC and BIC in model selection. Sociological Methods and Research.

[CR7] Chalmers RP (2012). mirt : A multidimensional item response theory package for the R environment. Journal of Statistical Software.

[CR8] Chen, W.-H., & Thissen, D. (1997). Local dependence indexes for item pairs using item response theory. *Journal of Educational and Behavioral Statistics Fall*, *22*(3), 265–289.

[CR9] Christensen KB, Makransky G, Horton M (2017). Critical values for Yen’s Q3: Identification of local dependence in the Rasch model using residual correlations. Applied Psychological Measurement.

[CR10] Craig, F., Tenuta, F., De Giacomo, A., Trabacca, A., & Costabile, A. (2021). A systematic review of problematic video-game use in people with autism spectrum disorders. *Autism Spectrum Disorders*, *82*.10.1016/j.rasd.2021.101726

[CR11] de Ayala RJ (2009). The theory and practice of item response theory.

[CR12] de Hesselle, L. C., Rozgonjuk, D., Sindermann, C., Pontes, H. M., & Montag, C. (2020). The associations between Big Five personality traits, gaming motives, and self-reported time spent gaming. *Personality and Individual Differences*, *November*, 110483. 10.1016/j.paid.2020.110483

[CR13] Desjardins CD, Bulut O (2018). Handbook of educational measurement and psychometrics using R. CRC Press.

[CR14] Dieris-Hirche, J., Pape, M., te Wildt, B. T., Kehyayan, A., Esch, M., Aicha, S., Herpertz, S., & Bottel, L. (2020). Problematic gaming behavior and the personality traits of video gamers: A cross-sectional survey. *Computers in Human Behavior*, *106*(July 2019), 106272. 10.1016/j.chb.2020.106272

[CR15] Donnellan MB, Oswald FL, Baird BM, Lucas RE (2006). The mini-IPIP scales: Tiny-yet-effective measures of the Big Five factors of personality. Psychological Assessment.

[CR16] Drasgow F, Levine MV, Williams EA (1985). Appropriateness measurement with polychotomous item response models and standardized indices. British Journal of Mathematical and Statistical Psychology.

[CR17] Embretson SE, Reise SP (2000). Item response theory for psychologists.

[CR18] ESA. (2020). Essential facts about the computer and video game industry. In *Social Science Computer Review* (Vol. 4, Issue 1). Retrieved December 21, 2020, from https://www.theesa.com/wp-content/uploads/2020/07/Final-Edited-2020-ESA_Essential_facts.pdf

[CR19] Europe’s Video Game Industry. (2019). *Key facts*. Retrieved December 21, 2020, from https://www.isfe.eu/isfe-key-facts/

[CR20] Evren C, Dalbudak E, Topcu M, Kutlu N, Evren B, Pontes HM, Dalbudak E, Evren B, Topcu M, Kutlu N (2020). Psychometric validation of the Turkish nine-item Internet Gaming Disorder Scale-Short Form (IGDS9-SF). Psychiatry Research.

[CR21] Fuster H, Carbonell X, Pontes HM, Griffiths MD (2016). Spanish validation of the Internet Gaming Disorder-20 (IGD-20) Test. Computers in Human Behavior.

[CR22] Goldberg LR (1992). The development of markers for the big-five factor structure. Psychological Assessment.

[CR23] Griffiths, M. D. (2019). The therapeutic and health benefits of playing video games. In A. Attrill-Smith, C. Fullwood, M. Keep, & D. J. Kuss (Eds.), *The Oxford handbook of cyberpsychology* (pp. 484–505). Oxford University Press. 10.1093/oxfordhb/9780198812746.013.27

[CR24] Hair, J. F., Black, W. C., Babin, B. J., & Anderson, R. E. (2010). Multivariate data analysis. A global perspective (seventh edition). In *Pearson Prentice Hall.* (Vol. 232). 10.1016/j.foodchem.2017.03.133

[CR25] Hu LT, Bentler PM (1999). Cutoff criteria for fit indexes in covariance structure analysis: Conventional criteria versus new alternatives. Structural Equation Modeling.

[CR26] International Test Commission. (2018). ITC guidelines for translating and adapting tests (second edition). *International Journal of Testing*, *18*(2), 101–134. 10.1080/15305058.2017.1398166

[CR27] Jöreskog, K. G. (1993). Testing structural equation models. In *Testing structural equation models* (pp. 294–316). Sage Publication.

[CR28] Jorgensen, T. D., Pornprasertmanit, S., Schoemann, A. M., & Rosseel, Y. (2020). *semTools: Useful tools for structural equation modeling. R package version 0.5–3*.

[CR29] Kang T, Chen TT (2008). Performance of the generalized S-X2 item fit index for polytomous IRT models. Journal of Educational Measurement.

[CR30] Kayiş AR, Satici SA, Yilmaz MF, Şimşek D, Ceyhan E, Bakioǧlu F (2016). Big five-personality trait and internet addiction: A meta-analytic review. Computers in Human Behavior.

[CR31] Kim DJ, Kim K, Lee HW, Hong JP, Cho MJ, Fava M, Mischoulon D, Heo JY, Jeon HJ (2017). Internet game addiction, depression, and escape from negative emotions in adulthood: A nationwide community sample of Korea. Journal of Nervous and Mental Disease.

[CR32] Király O, Sleczka P, Pontes HM, Urbán R, Griffiths MD, Demetrovics Z (2017). Validation of the Ten-Item Internet Gaming Disorder Test (IGDT-10) and evaluation of the nine DSM-5 Internet Gaming Disorder criteria. Addictive Behaviors.

[CR33] Király O, Bothe B, Ramos-Diaz J, Rahimi-Movaghar A, Lukavska K, Hrabec O, Miovsky M, Billieux J, Deleuze J, Nuyens F, Karila L, Griffiths MD, Nagygyörgy K, Urbán R, Potenza MN, King DL, Rumpf HJ, Carragher N, Demetrovics Z (2019). Ten-item internet gaming disorder test (IGDT-10): Measurement invariance and cross-cultural validation across seven language-based samples. Psychology of Addictive Behaviors.

[CR34] Lemmens JS, Valkenburg PM, Peter J (2011). The effects of pathological gaming on aggressive behavior. Journal of Youth and Adolescence.

[CR35] Li C-H (2016). Confirmatory factor analysis with ordinal data: Comparing robust maximum likelihood and diagonally weighted least squares. Behavior Research Methods.

[CR36] Li, H., Zou, Y., Wang, J., & Yang, X. (2016). Role of stressful life events, avoidant coping styles, and neuroticism in online game addiction among college students: A moderated mediation model. *Frontiers in Psychology*, *7*(NOV), 1794. 10.3389/fpsyg.2016.01794 10.3389/fpsyg.2016.01794 PMC5118950 27920734

[CR37] Liang L, Ren H, Cao R, Hu Y, Qin Z, Li C, Mei S (2020). The effect of COVID-19 on youth mental health. Psychiatric Quarterly.

[CR38] López-Fernández, F. J., Mezquita, L., Etkin, P., Griffiths, M. D., Ortet, G., & Ibáñez, M. I. (2020). The role of violent video game exposure, personality, and deviant peers in aggressive behaviors among adolescents: A two-wave longitudinal study. *Cyberpsychology, Behavior, and Social Networking*, *00*(00). 10.1089/cyber.2020.0030 10.1089/cyber.2020.0030 33252248

[CR39] Lovibond PF, Lovibond SH (1995). Manual for the depression anxiety stress scales.

[CR40] Maldonado-Murciano L, Pontes HM, Griffiths MD, Barrios M, Gómez-Benito J, Guilera G (2020). The Spanish version of the Internet Gaming Disorder Scale-Short Form (IGDS9-SF): Further examination using item response theory. International Journal of Environmental Research and Public Health.

[CR41] Mandryk RL, Frommel J, Armstrong A, Johnson D (2020). How passion for playing World of Warcraft predicts in-game social capital, loneliness, and wellbeing. Frontiers in Psychology.

[CR42] Männikkö N, Ruotsalainen H, Miettunen J, Pontes HM, Kääriäinen M (2020). Problematic gaming behaviour and health-related outcomes: A systematic review and meta-analysis. Journal of Health Psychology.

[CR43] Martínez-Molina A, Arias VB (2018). Balanced and positively worded personality short-forms: Mini-IPIP validity and cross-cultural invariance. PeerJ.

[CR44] Masters GN (1982). A Rasch model for partial credit scoring. Psychometrika.

[CR45] Mihara S, Higuchi S (2017). Cross-sectional and longitudinal epidemiological studies of Internet gaming disorder: A systematic review of the literature. Psychiatry and Clinical Neurosciences.

[CR46] Millsap RE (2011). Statistical approaches to measurement invariance (Vol. 66).

[CR47] Montag C, Schivinski B, Sariyska R, Kannen C, Demetrovics Z, Pontes HM (2019). Psychopathological symptoms and gaming motives in disordered gaming—A psychometric comparison between the WHO and APA diagnostic frameworks. Journal of Clinical Medicine.

[CR48] Moore, S., Satel, J., & Pontes, H. M. (2021). Investigating the role of health factors and psychological well-being in Gaming Disorder. *Cyberpsychology, Behavior, and Social Networking*. 10.1089/cyber.2021.0050 34788152

[CR49] Müller KW, Beutel ME, Egloff B, Wölfling K (2014). Investigating risk factors for internet gaming disorder: A comparison of patients with addictive gaming, pathological gamblers and healthy controls regarding the big five personality traits. European Addiction Research.

[CR50] Muraki E (1992). A generalized partial credit model: Application of an EM algorithm. ETS Research Report Series.

[CR51] Orlando M, Thissen D (2000). Likelihood-based item-fit indices for dichotomous item response theory models. Applied Psychological Measurement.

[CR52] Ostinelli EG, Zangani C, Giordano B, Maestri D, Gambini O, D’Agostino A, Furukawa TA, Purgato M (2021). Depressive symptoms and depression in individuals with internet gaming disorder: A systematic review and meta-analysis IDepressive symptoms and depression in individuals with Internet Gaming Disorder: A systematic review and meta-analysis. Journal of Affective Disorders.

[CR53] Paulus FW, Ohmann S, von Gontard A, Popow C (2018). Internet gaming disorder in children and adolescents: A systematic review. Developmental Medicine & Child Neurology.

[CR54] Pontes HM (2017). Investigating the differential effects of social networking site addiction and Internet gaming disorder on psychological health. Journal of Behavioral Addictions.

[CR55] Pontes, H. M. (2018). Making the case for video game addiction: Does it exist or not? In C. J. Ferguson (Ed.), *Video game influences on aggression, cognition, and attention* (pp. 41–57). Springer International Publishing. 10.1007/978-3-319-95495-0_4

[CR56] Pontes HM, Griffiths MD (2014). Assessment of internet gaming disorder in clinical research: Past and present perspectives. Clinical Research and Regulatory Affairs.

[CR57] Pontes HM, Griffiths MD (2015). Measuring DSM-5 internet gaming disorder: Development and validation of a short psychometric scale. Computers in Human Behavior.

[CR58] Pontes HM, Griffiths MD (2016). Portuguese validation of the Internet Gaming Disorder Scale-Short-Form. Cyberpsychology, Behavior, and Social Networking.

[CR59] Pontes, H. M., & Griffiths, M. D. (2020). A new era for gaming disorder research: Time to shift from consensus to consistency. *Addictive Behaviors*, *103*.10.1016/j.addbeh.2019.106059 10.1016/j.addbeh.2019.106059 31473045

[CR60] Pontes HM, Király O, Demetrovics Z, Griffiths MD (2014). The conceptualisation and measurement of DSM-5 internet gaming disorder: The development of the IGD-20 test. PLoS ONE.

[CR61] Pontes, H. M., Schivinski, B., Sindermann, C., Li, M., Becker, B., Zhou, M., & Montag, C. (2021). Measurement and conceptualization of gaming disorder according to the World Health Organization framework: The development of the gaming disorder. *International Journal of Mental Health and Addiction*, 1–21.10.1007/s11469-019-00088-z

[CR62] Poon LYJ, Tsang HWH, Chan TYJ, Man SWT, Ng LY, Wong YLE, Lin C-Y, Chien C-W, Griffiths MD, Pontes HM, Pakpour AH (2021). Psychometric properties of the Internet Gaming Disorder Scale-Short-Form (IGDS9-SF): A systematic review. Journal of Medical Internet Research.

[CR63] R Core Team. (2021). *R: A language and environment for statistical computing*. Retrieved April 21, 2021, from https://www.r-project.org/

[CR64] Rosseel, Y. (2012). lavaan: An R package for structural equation modeling. R package version 0.5–15. *Journal of Statistical Software*, *48*(2), 1–36.

[CR65] Rozgonjuk D, Schivinski B, Pontes HM, Montag C (2021). Problematic online behaviors among gamers: The links between problematic gaming, gambling, shopping, pornography use, and social networking. International Journal of Mental Health and Addiction.

[CR66] Şalvarlı Şİ, Griffiths MD (2019). Internet gaming disorder and its associated personality traits: A systematic review using PRISMA Guidelines. International Journal of Mental Health and Addiction.

[CR67] Samejima F (1999). General graded response model.

[CR68] Sánchez-Iglesias I, Bernaldo-De-Quirós M, Labrador FJ, Estupiñá Puig FJ, Labrador M, Fernández-Arias I (2020). Spanish validation and scoring of the Internet Gaming Disorder Scale - Short-Form (IGDS9-SF). Spanish Journal of Psychology.

[CR69] Sass DA, Schmitt TA, Marsh HW (2014). Evaluating model fit with ordered categorical data within a measurement invariance framework: A comparison of estimators. Structural Equation Modeling: A Multidisciplinary Journal.

[CR70] Severo RB, Barbosa APPN, Fouchy DRC, da C Coelho FM, Pinheiro RT, de Figueiredo VLM, de Siqueira Afonso V, Pontes HM, Pinheiro KAT (2020). Development and psychometric validation of Internet Gaming Disorder Scale-Short-Form (IGDS9-SF) in a Brazilian sample. Addictive Behaviors.

[CR71] Sherry JL (2001). The effects of violent video games on aggression a meta-analysis. Human Communication Research.

[CR72] Sinharay S, Haberman SJ (2014). How often is the misfit of item response theory models practically significant?. Educational Measurement: Issues and Practice.

[CR73] Snodgrass JG, Lacy MG, Dengah F, Eisenhauer S, Batchelder G, Cookson RJ (2014). A vacation from your mind: Problematic online gaming is a stress response. Computers in Human Behavior.

[CR74] Stavropoulos V, Adams BLM, Beard CL, Dumble E, Trawley S, Gomez R, Pontes HM (2019). Associations between attention deficit hyperactivity and internet gaming disorder symptoms: Is there consistency across types of symptoms, gender and countries?. Addictive Behaviors Reports.

[CR75] Wittek CT, Finserås TR, Pallesen S, Mentzoni RA, Hanss D, Griffiths MD, Molde H (2016). Prevalence and predictors of video game addiction: A study based on a national representative sample of gamers. International Journal of Mental Health and Addiction.

[CR76] World Health Organization. (2019). *International classification of diseases for mortality and morbidity statistics (11th Revision).* Retrieved December 21, 2020, from https://icd.who.int/browse11/l-m/en

[CR77] Wright B, Panchapakesan NA (1969). A procedure for sample free item analysis. Educational and Psychological Measurement.

[CR78] Yam CW, Pakpour AH, Griffiths MD, Yau WY, Lo CLM, Ng JMT, Lin CY, Leung H (2019). Psychometric testing of three Chinese online-related addictive behavior instruments among Hong Kong university student. Psychiatric Quarterly.

[CR79] Zhao Y (2017). Impact of IRT item misfit on score estimates and severity classifications: An examination of PROMIS depression and pain interference item banks HHS Public Access. Quality of Life Research.

