# Supplementary material for: Psychometric Validation of the Spanish Gaming Disorder Test (GDT): Item Response Theory and Measurement Invariance Analysis
Source: Int J Ment Health Addict. 2021 Dec 6:1–19. Online ahead of print. doi: 10.1007/s11469-021-00704-x (PMC8647963; doi:10.1007/s11469-021-00704-x)
Supplement: Supplementary file 1 — Supplementary file1 (DOCX 94 KB) [file 11469_2021_704_MOESM1_ESM.docx]

# Supplementary material

Figure S1


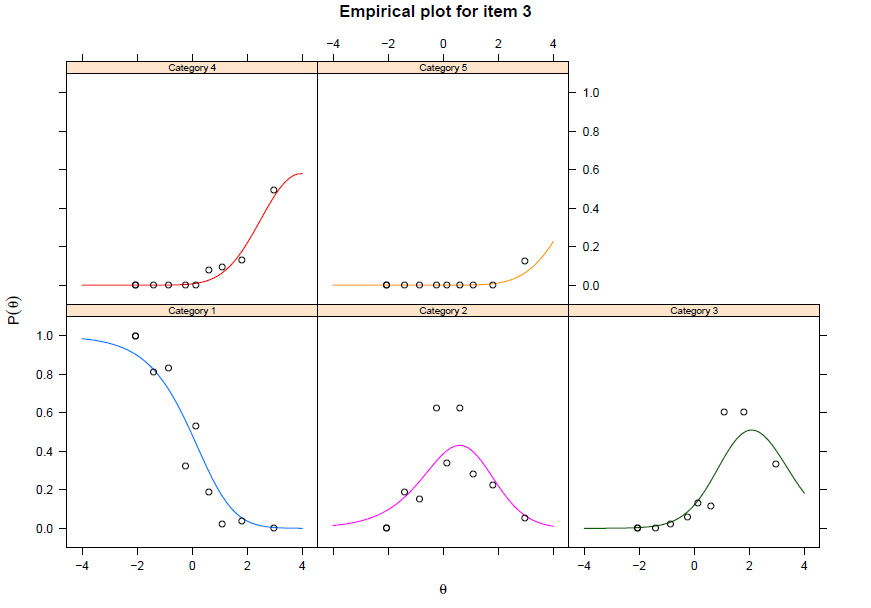


Empirical plot of item 3 of the Gaming Disorder Test (GDT)

Figure S2


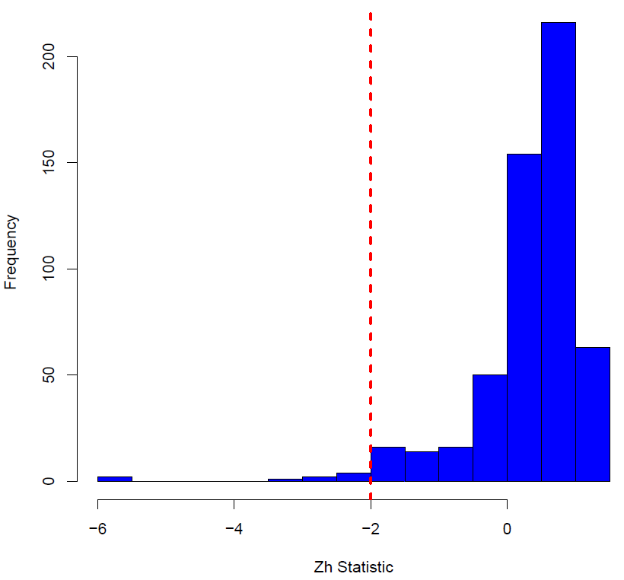


*Histogram of person fit Z_h_ statistic (Drasgow et al., 1985)*
